# The role of music in ADHD: A multi-dimensional computational and theoretical analysis

**DOI:** 10.1371/journal.pone.0324369

**Published:** 2025-08-01

**Authors:** Krishnashree Achuthan, Sugandh Khobragade

**Affiliations:** Center for Cybersecurity Systems and Networks, Amrita Vishwa Vidyapeetham, Amritapuri, Kollam, Kerala, India; Istituto di Fisiologia Clinica Consiglio Nazionale delle Ricerche, ITALY

## Abstract

Attention-Deficit Hyperactivity Disorder (ADHD), a neurodevelopmental disorder affecting children and adults worldwide, has seen a significant rise in diagnoses and medication prescriptions in recent decades. This trend has emphasized the need for non-pharmacological interventions such as music to aid ADHD management. This study explores the musical experiences of individuals with ADHD through a comprehensive analysis of user-generated content from the Reddit r/ADHD community between 2014–2024. Advanced computational techniques, including large language models such as Gemini 1.5 Pro and LLAMA 3.1 were employed for data extraction and categorization. Additionally, APIs from digital streaming platforms were utilized to analyze musical characteristics and lyrical content of 9,215 tracks across three distinct categories: focus music, stuck songs, and general purpose. Insights from selective attention, emotion arousal and mood congruence theories were used to interpret the findings. Statistical analysis revealed significant variations in musical characteristics, with instrumentalness showing the largest effect size across contexts, suggesting unique musical preferences among individuals with ADHD. Correlation analyses uncovered complex interrelationships between musical attributes, particularly in focus music, where energy, speechiness, and instrumental characteristics displayed distinctive patterns. The sentiment and popularity analysis of lyrics further illuminated the emotional landscape of music in ADHD experiences, revealing a strategic approach to musical selection as a potential cognitive and emotional self-regulation mechanism.

## 1. Introduction

ADHD, or Attention Deficit Hyperactivity Disorder, is a neurodevelopmental condition that affects an estimated 5% to 7% of children and around 2.5% of adults worldwide [1]. What is particularly concerning is the sharp rise in ADHD diagnoses and prescription rates for ADHD medications over the past few decades, reaching up to 50-fold in some countries [2]. This dramatic escalation raises important questions about diagnosis practices, treatment approaches, and the broader societal factors contributing to this trend. ADHD is characterized by symptoms such as inattention, hyperactivity, and impulsivity, which can affect cognitive functioning and emotional regulation [3]. Individuals with ADHD face significant challenges across various aspects of life. They are markedly more likely to experience emotional, conduct, and peer problems [4]. ADHD individuals also have impairments in selective attention, sustained attention, response precision, cognitive flexibility, and working memory [5]. Additionally, ADHD is a highly heterogeneous condition, making it challenging to diagnose and treat effectively. Cognitive challenges are also notable, including moderate to small difficulties in abstract problem-solving, various types of memory, and attention spans [6]. Furthermore, ADHD correlates with higher instances of obesity and diabetes [7]. There is also a moderate link between ADHD and sleep disorders. These multifaceted challenges not only diminish quality of life but also lead to significant psychosocial difficulties [8], increase the likelihood of accidental injuries, and can even result in premature death. Consequently, managing ADHD incurs substantial economic costs globally, amounting to billions of dollars annually [9].

Given these extensive challenges, there is a growing interest in non-traditional approaches to support ADHD management [10]. Beyond conventional therapies, emerging research suggests that music might play a pivotal role in managing ADHD symptoms. Studies have shown that individuals with ADHD often use music as a non-pharmacological tool to help regulate their attention and impulsivity [11]. For instance, music with certain audio characteristics—like tempo, energy, and rhythm—has been associated with increased focus and reduced hyperactivity [12]. To better understand the mechanisms by which music influences attention and emotional states, several psychological theories offer useful perspectives. Emotional arousal theory, for example, suggests that emotionally stimulating experiences can enhance memory recall, making it easier to retain focus. Selective attention theory highlights how focusing on specific stimuli, like rhythmic or familiar music, can help filter out distractions and sustain attention on a given task [13]. Additionally, mood congruence theory [14] proposes that individuals tend to recall information congruent with their current emotional state, shedding light on how music might support emotional regulation [15]. Together, these theories illustrate the cognitive and emotional impact of music, suggesting a potential psychological basis for its benefits in ADHD management.

### 1.1 Related works

#### 1.1.1 Music’s dual role: engagement and healing.

The developing field of music research has shown its profound impact across general and therapeutic contexts, revealing how deeply music influences emotional, cognitive, and social dynamics. [16] investigate how music chosen for studying and sleeping shares common features despite the different arousal requirements for each activity. The researchers find that both study and sleep music playlists often feature tracks with calming attributes, low complexity, and stable tonality, aimed at minimizing distractions. This similarity suggests that curators and listeners prioritize music that creates a non-intrusive auditory environment, facilitating either concentration or relaxation and enabling themselves towards selective attention. [17] employ machine learning techniques to analyze how musical features like loudness, danceability, and instrumentalness predict song popularity across various genres. Their findings highlight that danceability and genre significantly impact a song’s commercial success, indicating that listeners prefer music that is not only appealing but also encourages physical and emotional engagement. [18] delve into the social and emotional motivations behind music choices for dancing, categorizing dance music based on audio features that align with listeners’ needs for emotional regulation and social interaction. Their findings highlight the significant role of dance music in going beyond mere physical activity to encompass crucial aspects of emotional and social experiences. In the realm of medical interventions, particularly for pain management and mental health, the role of music is increasingly being recognized not just as a supplementary treatment but as a critical element that enhances patient outcomes. [19] explore how self-selected music plays a crucial role in analgesic music listening interventions. Patients often choose music with high energy, danceability, and lyrics—characteristics that likely make the music more engaging and effective at managing pain compared to the slower, non-lyrical music typically chosen by experimenters. This preference aligns with findings by [20], who demonstrated the impact of tempo and genre on physiological arousal, reinforcing insights from emotions arousal theory. [21] examine the musical preferences of individuals with depression, revealing a tendency towards music with higher energy and instrumentalness. This study illustrates music’s therapeutic potential to modulate mood and provide emotional support. It also discusses the possible echo chamber effect, where the chosen music could reinforce specific emotional states.

#### 1.1.2 Social media reflecting lived experiences.

Social media is a well-established platform that plays a central role in disseminating information and shaping how individuals communicate. More recently, it has become instrumental in providing insights into personal perspectives on health, illnesses, and treatment. Platforms like Twitter, Instagram, and Facebook, alongside specialized forums, provide individuals with chronic illnesses, mental health challenges, and other medical conditions a space to share their stories, symptoms, and treatment experiences [22]. This sharing fosters a sense of community, reduces isolation, and empowers individuals by validating their experiences through collective understanding [23]. Additionally, by engaging with peers facing similar challenges, users build robust support networks, access valuable coping strategies, and benefit from the emotional and practical guidance offered by others navigating similar paths [24].

Beyond community support, social media insights can benefit healthcare providers by shedding light on patient concerns and lived experiences, enhancing patient education, and improving healthcare outcomes [25]. Insights gathered from these platforms reveal real-world patient experiences, helping providers address concerns that may be overlooked in traditional clinical settings. These platforms also facilitate identity management, social participation, and patient activism [26], helping individuals align their health experiences with their personal identities and contributing to broader advocacy movements, such as #MentalHealthAwareness.

Moreover, patient-driven data from social media provides valuable perspectives for researchers and healthcare professionals [27]. These insights can inform clinical practices, especially regarding issues like medication adherence, treatment side effects, and overall patient satisfaction. For healthcare providers, understanding patterns in patient experiences shared online can improve communication, tailor educational materials, and even address misconceptions [28]. The potential to analyze trends in symptoms or quality of life across patient communities can lead to more responsive, patient-centered care. Thus, social media offers a unique, patient-driven lens into the medical world, providing a valuable complement to clinical perspectives and creating a dynamic space for collective patient insight, support, and advocacy. Online communities like Reddit offer a rich source of data for understanding the lived experiences of individuals with ADHD. Subreddits such as r/ADHD function as informal support networks where users share coping strategies, including their use of music for symptom management.

#### 1.1.3 Music and ADHD.

Several studies collectively indicate the promising role of music therapy in managing ADHD symptoms, offering insights into various cognitive and emotional benefits. [29] explored listening therapy, highlighting potential improvements in attention and behavioral issues among adolescents with ADHD. While limited to three case studies, their findings suggest that auditory and vestibular training could be a complementary approach for symptom management. Complementing this, [30] demonstrated that music and movement therapy improved attention and quality of life in children with ADHD, supported by EEG findings showing enhanced brain activity patterns associated with attentional focus.

[31] provide an emotional dimension, showing that Mozart’s compositions can positively affect mood in adults with ADHD, presenting an accessible, non-pharmacological option for emotional regulation. [32] further examined music’s impact on emotional well-being, revealing that music therapy led to increased serotonin levels and reduced stress markers in children and adolescents with ADHD, pointing to its potential for managing ADHD-related emotional dysregulation. [33] delved into the neurocognitive aspect, noting that different musical compositions can distinctly influence brainwave patterns in children with ADHD, suggesting tailored music types could enhance attention. However, as [34] highlight, the benefits of music are multifaceted; while it aids attention for those with ADHD, it may also have a tendency to distract, indicating a need for customized interventions. Collectively, these findings support music therapy’s integration into ADHD treatment plans, although further research is essential to validate these effects across larger populations and diverse musical contexts.

Studies have shown that individuals with ADHD often use music as a non-pharmacological tool to help regulate their attention and impulsivity [11]. For instance, music with certain audio characteristics—like tempo, energy, and rhythm—has been associated with increased focus and reduced hyperactivity [12]. Research by [21] found that ADHD users tend to gravitate towards specific genres or types of music based on their need for stimulation or emotional regulation. However, fewer studies have examined how specific audio features in songs affect ADHD-related contexts such as focus music, preferred tracks and so on.

Music has long been associated with emotional expression, and sentiment analysis of song lyrics has become a useful tool for understanding how lyrical content resonates with listeners. In the context of ADHD, emotional engagement with music may be particularly important, as emotional dysregulation is a core symptom of the disorder [35]. Existing studies have explored the role of lyrical sentiment in shaping user preferences, particularly in mood regulation. However, there is limited research on how the sentiment of lyrics relates to the popularity of songs among ADHD individuals. Understanding this relationship is crucial, as it may offer insights into the kinds of emotional messages that resonate with listeners in ADHD-related music categories. Excessive mind wandering and rumination are often observed in individuals with ADHD [36], and music, particularly songs that get “stuck” in one’s head, may play a role in reinforcing these patterns. Studies on “earworms” (songs that persistently replay in the mind), or involuntary music imagery [37,38] have shown that certain structural features of songs, such as lyrical repetition, play a critical role in making a song memorable or “sticky” [39]. However, research is lacking on how lyrical repetition specifically affects ADHD individuals and how it contributes to the experience of “stuck songs” in ADHD contexts.

In spite of growing recognition of music’s role in ADHD management, existing research tends to focus on structured experimental settings, leaving a gap in understanding how individuals with ADHD use music in real-world, everyday contexts. This study addresses several gaps at the intersection of ADHD and music. By analyzing user discussions on the r/ADHD subreddit, with a focus on how different music contexts—focus music, stuck songs, and general-purpose music—affect individuals with ADHD, the following research questions are explored in this study. Neutral music refers to songs that are neither associated with focus nor reported as stuck songs.

1. How do the audio features of songs differ across ADHD-related music contexts (i.e., general purpose music, focus music, and stuck songs), and what might these differences suggest about music preferences or effects in individuals with ADHD?
2. What relationships exist between audio features in ADHD-related music contexts, and how do these correlations differ across general purpose music, focus music, and stuck songs categories?
3. How does the sentiment of song lyrics relate to song popularity in ADHD-related music contexts, and are there notable differences in this relationship across general purpose music, focus music, and stuck songs categories?

The unique contributions of this work include Firstly, this study provides a novel exploration of the relationship between music and ADHD symptom management by analyzing user-generated content from the Reddit r/ADHD community over a ten-year period. The posts and comments from this community provide qualitative and quantitative data, offering a window into how individuals with ADHD perceive and use music in their daily lives. This unique methodological approach captures authentic insights into how individuals with ADHD use music to manage cognitive and emotional challenges. Secondly, the study employs advanced computational tools, including large language models like Gemini 1.5 Pro and LLAMA 3.1, for precise data extraction and categorization. APIs from digital streaming services like Spotify and Genius, were used to extract and analyze both audio and lyrical features of music. This multi-dimensional approach allowed for a robust and scalable analysis of over 9,200 tracks. Thirdly, the categorization of musical experiences into focus music, stuck songs, and general purpose types, along with the analysis of their distinct audio feature profiles, represents a significant advancement in understanding the preferences and needs of individuals with ADHD. Fourthly, the correlation analysis of audio features offers new perspectives on how individuals with ADHD respond to different musical attributes. Fifthly, the integration of emotional arousal, selective attention, and mood congruence theories to interpret findings bridges theoretical frameworks with empirical evidence. Sixthly, the sentiment and popularity analysis of song lyrics provides unique insights into the emotional landscape of music preferences within ADHD contexts. Lastly, this study contributes to the broader field by offering practical applications across multiple domains, including therapy, education, and technology.

## 2. Methodology

**Fig 1:**
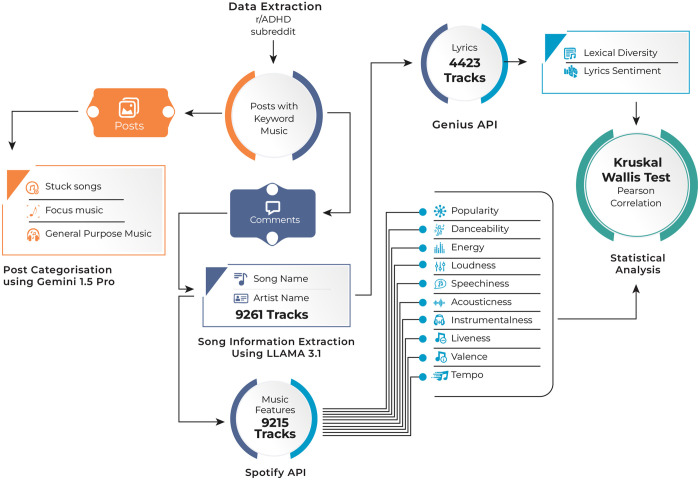
Research methodology: data extraction and analysis phases.

The methodology for this study, as depicted in Fig 1, encompasses data extraction and text analysis phases. The selection of Reddit as a research platform is grounded in its unique methodological advantages for capturing user-generated insights into complex psychological phenomena. Emerging research has increasingly validated Reddit as a robust source of qualitative data, demonstrating its potential for investigating mental health-related experiences across various domains. Recent studies, such as [40] analysis of women’s self-disclosure in infertility communities and [41] examination of suicide support communities, have demonstrated the platform’s capacity to provide rich insights into sensitive psychological experiences. The platform’s value for analyzing public discourse and user engagement has been further validated by [42,43] in their investigation of cybersecurity discussions.

### 2.1 Data collection and preprocessing

The data for this study was obtained from the Reddit Pushshift archive, specifically the repository available at https://the-eye.eu/redarcs/ [44]. The posts and comments data for subreddit r/ADHD were downloaded in ZST format and subsequently converted into CSV format for the analysis. The raw dataset consisted of 6,42,683 posts and 54,16,531 comments spanning the years 2011–2022. Metadata included timestamps, post/comment bodies, author usernames (anonymized for analysis), upvote counts, and submission titles where applicable. All posts and comments analyzed were in English. As Reddit does not provide user-level geographic information, we assume that most contributors are from English-speaking countries, consistent with the language and primary user base of the platform.

The collection and analysis of this data were conducted in compliance with Reddit’s terms of service and API usage policies at the time of retrieval. Since the dataset consists of publicly available posts and comments with no private or deleted content included, this study adheres to ethical guidelines for research using publicly accessible online data. User identifiers were anonymized during processing to protect privacy, and no attempts were made to link data to individual identities. Additionally, the Pushshift archive operates under fair-use principles for academic research, as outlined in its documentation.

The dataset underwent several rigorous cleaning and preprocessing steps to prepare it for analysis. First, post titles which had been removed or deleted were discarded, and any special characters or emoji present in titles were removed to ensure text consistency. Since the focus of this study is on user data, the post titles which contained external links were excluded and only user posts were retained. Next, posts with fewer than 5 comments were removed to maintain a threshold of engagement. As a final filtering step, only posts containing “music”, “Song”, or “songs” were selected, yielding 1,943 music-related posts. These posts were then merged with corresponding comments data. The comments were cleaned in a similar fashion to the posts by discarding removed or deleted comments and removing special characters and emojis from comments. Duplicate comments and posts were also removed to retain unique posts and comments. For the analysis, only the necessary variables were retained, which included the following: post ID, comment ID, post upvote score, comment upvote score, post title, post content, comment body, number of comments, postdate, and comment date.

### 2.2 Categorization of posts using Gemini 1.5 pro

A manual content analysis was conducted on a representative sample of r/ADHD posts to guide the development of coding categories for subsequent analysis. Leveraging the advanced natural language understanding capabilities of large language model Gemini 1.5 Pro [45], which demonstrates remarkable contextual comprehension and interpretation of text, a structured categorization approach was developed. Similarly, computational approaches are being used to understand ADHD through the analysis of various data types [46]. The categorization framework distinguished between: (1) focus music – titles indicating music used for concentration and productivity, (2) stuck songs – titles describing repetitive or intrusive musical experiences, and (3) general purpose – titles not falling into the above two primary musical experience categories.

Based on the initial analysis, a prompt for Gemini 1.5 Pro was crafted to systematically categorize these posts. An iterative prompt refinement process was employed, with each iteration involving manual checking of the categories and post titles. This approach allowed for progressive improvement of the categorization methodology, ensuring a more accurate classification of the musical experiences described in the r/ADHD community. The prompt explicitly instructed the model to objectively categorize each title based on the predefined labels, taking into account the subtle linguistic cues that might indicate different types of musical interaction in ADHD.

### 2.3 Song Information Extraction (SIE)

To extract song and artist names from user comments, LLAMA 3.1 [47] was employed, outperforming traditional Named Entity Recognition (NER) methods such as SpaCy, NLTK, and Flair Python libraries. These traditional methods struggled to handle the informal language and subtle ways in which users referred to music in comments. LLAMA 3.1, with its advanced contextual understanding and ability to process complex user-generated content, was better suited for extracting music-related entities with greater accuracy and flexibility. This highlights the increasing capability of advanced AI models in handling complex, real-world data, a trend observed in the broader development of artificial general intelligence [48].

Traditional NER methods often face challenges in user-generated contexts due to slang, abbreviations, and varied phrasing. In contrast, LLAMA 3.1 leverages large-scale pre-trained models capable of understanding such intricacies [49]. The GROQ API [50] was used to access the LLAMA3-8b-8192 model for this task. The model was prompted to identify and extract both song and artist names, even when multiple entities appeared within the same comment. For comments where no entities were found, the model returned “none.”

To ensure compliance with GROQ’s token limits, a rate limiter, and a batch size of 30 comments were implemented during the extraction process. The output was formatted in JSON, containing the original comment, song name, and artist name. This structured output was then seamlessly integrated into the dataset for further analysis. Using this approach, Song Information Extraction (SIE) successfully identified 9,261 tracks, significantly enhancing the dataset’s utility for subsequent analyses.

### 2.4 Song features extraction

Using track information (song and artist names), the Spotify API [51] was employed to extract detailed audio features for each track, providing a rich dataset for analysis. These features included the duration of the track in milliseconds, a binary variable indicating whether the track contains explicit language, and valence, which measures the musical positiveness conveyed by the track, with higher values representing more positive or happy tunes. Additional features extracted were tempo, measured in beats per minute (BPM), instrumentalness, indicating the likelihood that a track is instrumental, and popularity based on user engagement and streaming activity. The dataset also included features such as danceability, which assesses the track’s suitability for dancing by considering rhythm stability and beat strength, key, representing the musical key, and mode, which specifies whether the track is in a major or minor key. other audio characteristics included speechiness, reflecting the presence of spoken words; loudness, the average volume in decibels; acousticness, the degree to which the track sounds acoustic; liveness, indicating the presence of an audience; and genres associated with the artist. These audio features provide valuable insights into the musical and emotional characteristics of the tracks, enabling an in-depth analysis of their traits. Audio features for 9,215 tracks were successfully extracted using the Spotify API.

In addition to the audio features, lyrics were obtained using the Genius API [52] and subjected to a rigorous cleaning process to ensure the data was suitable for analysis. This process began by removing unwanted phrases at the beginning of the lyrics, such as contributor annotations like “1 Contributor” or “11 Contributors,” and instances of the word “lyrics” paired with the track name. Text in square brackets, often used for annotations such as “[Chorus]” or “[Verse],” was removed both at the beginning and throughout the lyrics. Promotional phrases, such as “See...Live” or “You might also like,” were filtered out, along with trailing patterns like “embed” found at the end of some lyrics. To improve readability, special characters were stripped, extra spaces were eliminated, and the text was reformatted. Finally, duplicate entries were identified and removed based on the combination of song and artist names, ensuring that each track’s lyrics were uniquely represented in the dataset. This comprehensive cleaning process resulted in a structured and high-quality lyrical dataset. Using the Genius API, lyrics for 4,423 tracks were successfully obtained.

### 2.5 Statistical analysis

To explore the lyrical and musical characteristics of each track, several statistical analyses were conducted. Two key textual features were derived from the lyrics: lexical diversity and repeatability. Lexical diversity was quantified using the Type-Token Ratio (TTR), a widely used metric in text analysis [53]. It is calculated as:


TTR = Number of Unique words/ Toral words

This metric evaluates the variety of unique words in relation to the total word count, providing a measure of linguistic richness. Repeatability, a complementary feature, was calculated as 1 − TTR, representing the extent of word repetition in the lyrics. These features offer insight into the lyrical complexity and redundancy of the tracks.

In addition to lexical features, sentiment analysis of the lyrics was performed using the *syuzhet* library in R [54]. The lyrics were tokenized, and sentiment scores were assigned to individual words based on the Bing sentiment lexicon, which is a reliable resource for word-level sentiment classification. The overall sentiment of each track’s lyrics was computed by summing the sentiment scores of all tokens. The sentiment scores were normalized to fall within a range of −1–1, where −1 indicates the most negative sentiment, and 1 indicates the most positive sentiment.

**Fig 2:**
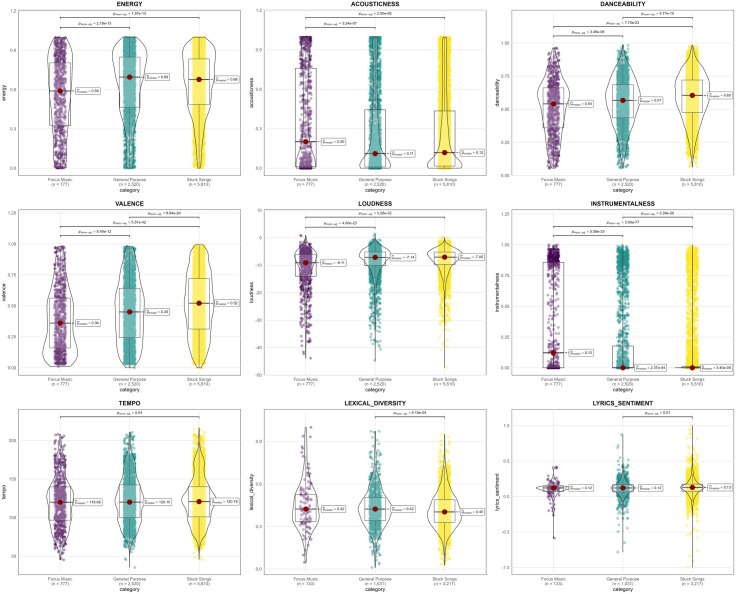
Analysis of audio and lyrical features across three music categories in r/ADHD.

To evaluate differences in audio features across topic groups, Analysis of Variance (ANOVA) was initially considered [55]. However, after examining the data distributions (Fig 2), it was observed that several variables did not meet ANOVA’s assumptions of normality and homogeneity of variance. Therefore, the non-parametric Kruskal-Wallis test was applied, which does not require these assumptions and is more appropriate for the data characteristics. The analysis was conducted using the *ggstatsplot* library in R [56], enabling both statistical computation and comprehensive visualizations. Where significant differences were identified, Dunn’s post-hoc test with Holm’s correction method was used to control family-wise error rates and pinpoint specific group differences. Effect sizes were calculated as epsilon-squared (ε²ordinal) values, with interpretations based on Cohen’s guidelines [57], where values around 0.01 indicate small effects, 0.06 medium effects, and 0.14 large effects.

Finally, Pearson correlation analysis was used to examine the relationships between audio features, lyrical features (lexical diversity and repeatability), and sentiment across different topic groups. Following Cohen’s guidelines [57], correlations are interpreted consistently as small (r = 0.1), medium (r = 0.3), and large (r = 0.5).

## 3. Result

### 3.1 Categories in r/ADHD

As shown in Table 1, the categorization revealed distinct patterns of musical experiences among individuals with ADHD. The focus music category, with 208 posts, illustrated how individuals use music as a strategy for concentration and task management. The stuck songs category, comprising 224 posts, captured the phenomenon of repetitive musical thoughts frequently reported in ADHD. The largest category, general purpose, with 503 posts, demonstrated the broad and varied musical discussions within the ADHD community.

**Table 1:** Post distribution and representative titles across music-related discussion categories.

Category	Number of Posts	Sample Post Titles
focus music	208	• “music to Listen to While focus” • “Does music help you get stuff done?” • “Desperately in need of music for studying focusing”
stuck songs	224	• “What song is currently playing in the background of your head?” • “Constant Internal music” • “What’s your current repeat this until I get bored of it song today”
general purpose	503	• “What’s your dopamine song for this month?” • “songs that remind you of ADHD” • “Looking for music recommends post-diagnosis medication”

### 3.2 ANOVA and comparative analysis

Fig 2 presents a detailed comparison of nine song features across the general purpose, focus music, and stuck songs topics, utilizing violin plots overlaid with box plots to demonstrate both distribution patterns and central tendencies. Kruskal-Wallis tests revealed significant differences across all features (Table 2).

**Table 2:** Kruskal-Wallis test results for musical and Lyrical Features.

Feature Name	𝛘2-Kruskal-wallis	p-value	𝛆2ordinal	CI 95%
Instrumentalness	381.69	1.31e-83	0.04	[0.04, 1.00]
Valence	245.58	4.95e-54	0.03	[0.02, 1.00]
Danceability	137.16	1.65e-30	0.02	[0.01, 1.00]
Energy	61.19	5.17e-14	6.72e-03	[4.73e-03, 1.00]
Acousticness	28.95	5.17e-07	3.18e-03	[1.20e-03, 1.00]
Loudness	141.89	1.55e-31	0.02	[0.01, 1.00]
Tempo	6.32	0.04	6.94e-04	[1.93e-04, 1.00]
Lexical Diversity	17.12	1.91e-04	3.91e-03	[1.90e-03, 1.00]
Lyrics Sentiment	10.44	5.42e-03	2.38e-03	[9.35e-04, 1.00]

By examining the shape and spread of the violin plots, we can observe several key differences in the distribution of variables among the topics. The focus music category tends to have a more distinct profile, with higher concentrations around certain musical attributes. For example, the focus music category exhibits a more concentrated distribution of positive valence, lower loudness, and greater instrumentalness compared to the general purpose and stuck songs categories. In contrast, the stuck songs category stands out with higher concentrations of energy, acousticness, and danceability. Looking at the median values further reinforces these observations. The stuck songs have the highest median energy, acousticness, and danceability, suggesting they tend to be the most energetic, acoustically driven, and danceable. The focus music, on the other hand, has the highest median valence and instrumentalness, indicating it contains the most positively valanced and instrument-focused music. The general purpose category generally falls between the focus music and stuck songs in terms of the median values, exhibiting a more balanced profile across the various features. However, it also shows wider distributions, suggesting more variability in the musical and lyrical characteristics within this category.

The comparison of song features across the three topics revealed statistically significant differences, as shown in Table 2. All examined characteristics displayed statistical significance, with varying degrees of strength. The analysis focused on effect sizes, measured by ε²ordinal, which represents the proportion of variance in the dependent variable accounted for by the independent variable. As illustrated in Fig 2, Instrumentalness showed the highest effect size among the analyzed features (*ε²ordinal = .04, p < .001*), though still within the small-to-medium range. This indicates that Instrumentalness exhibits the most pronounced variation across the three topics. Valence followed with an effect size of ε²ordinal = .03 (*p < .001*), suggesting differences in the emotional qualities of the music between topics. Danceability and Loudness both exhibited effect sizes of ε²ordinal = .02 (*p < .001*), indicating differences across topics. Energy and Acousticness demonstrated smaller effect sizes *(ε²*ordinal = 6.72e-03 and 3.18e-03 respectively, p < .001),** while still contributing to the differentiation between topics.

The remaining features showed the smallest effect sizes: Lexical Diversity (*ε²ordinal = 3.91e-03, p < .001*), Lyrics Sentiment (*ε²ordinal = 2.38e-03, p < .01*), and Tempo (*ε²ordinal = 6.94e-04, p = .04*). The confidence intervals for all effect sizes ranged from their specific lower bounds to 1.00, indicating some variability in the estimates.

### 3.3 Correlation analysis

**Fig 3:**
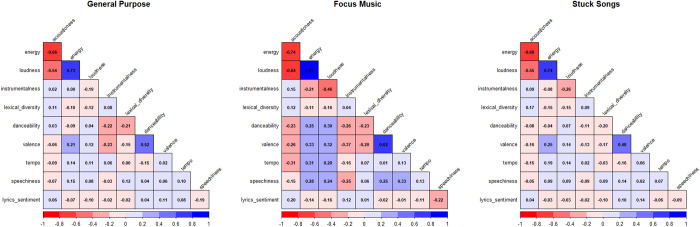
Pairwise correlations and distributions of audio and lyrical features across music categories.

Fig 3 presents a pair plot that visualizes the correlations between various audio features across the three categories: general purpose music, focus music, and stuck songs. The pair plot provides a comprehensive overview of the bivariate relationships between the features, allowing for a deeper understanding of the underlying patterns.

The correlation analysis reveals distinct patterns of musical attributes across ADHD-related contexts. Across all categories, two robust relationships emerge: a large positive correlation between energy and loudness (r ≈ 0.74 in all categories) and a medium-to-large correlation between danceability and valence (ranging from r = 0.41 in general purpose to r = 0.56 in focus music).

Beyond these similarities, the categories exhibit notable differences. Focus music displays a unique small positive correlation between energy and danceability (r = 0.12), contrasting with the absence of correlation in both general purpose and stuck songs categories (both r = −0.05). This suggests that energetic music becomes more danceable specifically in focus contexts.

The relationship between valence and instrumentalness also varies across categories. Although all correlations fall within the small effect size range, focus music shows the strongest negative correlation (r = −0.28), followed by general purpose (r = −0.21) and stuck songs (r = −0.16). This indicates that instrumental pieces in focus contexts tend to have less positive emotional valence.

Speechiness correlations further differentiate the categories. Focus music exhibits the strongest relationship between energy and speechiness (r = 0.23), compared to general purpose (r = 0.11) and stuck songs (r = 0.09), suggesting that vocal elements contribute more to perceived energy in focus contexts.

Similarly, tempo relationships show category-specific patterns, with focus music demonstrating the strongest correlation between energy and tempo (r = 0.24), compared to the overall sample (r = 0.18). This suggests faster tempos may be more integral to energy perception in focus music.

Lexical diversity and lyric sentiment correlations, while small in magnitude, reveal additional distinctions. Stuck songs show the strongest relationship between lexical diversity and energy (r = −0.14), while focus music exhibits a unique positive correlation between acousticness and lyrics sentiment (r = 0.19). These subtle differences highlight how linguistic features interact differently with musical elements across listening contexts.

### 3.4 Sentiment and popularity of lyrics across topics

**Fig 4:**
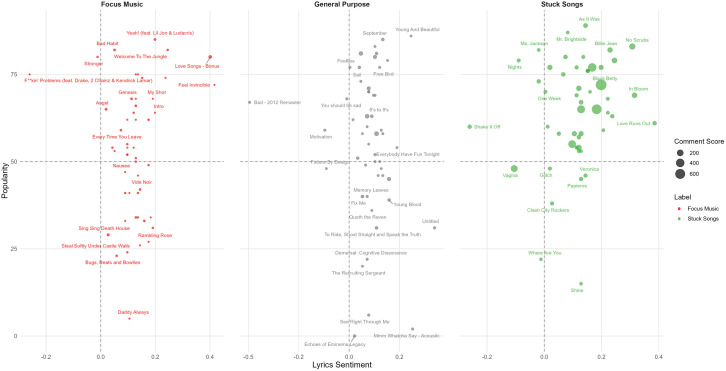
Lyrics sentiment vs song popularity: Top 50 most upvoted songs per music category.

Fig 4 presents a scatter plot comparing lyrics sentiment and popularity across three topics: focus music, general purpose, and stuck songs. The plot visualizes the top 50 songs per topic based on upvote scores, with lyrics sentiment on the x-axis and popularity on the y-axis. A horizontal line at 50 popularity and a vertical line at 0 sentiment divide the plot into quadrants, facilitating comparison across topics and sentiment-popularity relationships.

The distribution of songs across the plot reveals distinct patterns for each topic. focus music (red) tends to cluster more towards positive sentiment, with varying popularity levels, hinting at a preference for more upbeat or neutral lyrical content in music used for concentration. general purpose (blue) shows a wider spread, with songs distributed across all quadrants, indicating a diverse range of sentiment and popularity combinations. stuck songs (green) display a slight bias towards positive sentiment, with many highly popular tracks, suggesting that songs that become “earworms” are often both well-known and emotionally positive or neutral [38]. The distribution of stuck songs (green) in the figure reveals that many of these highly popular “earworm” tracks have received considerable upvote scores compared to the general purpose two topics.

Quadrant analysis provides further insights. The upper right quadrant (positive sentiment, high popularity) contains many stuck songs, while the upper left (negative sentiment, high popularity) features several general purpose songs. This distribution suggests that popular “earworms” often have positive lyrical content, while some popular music discussed in other contexts may have more negative or complex emotional themes. The lower quadrants show a mix of songs from all categories, with general purpose having a notable presence in the lower right (positive sentiment, lower popularity).

Notable outliers include “As It Was” by Harry Styles in the stuck songs category, standing out as a highly popular song with positive sentiment. These outliers highlight the unique characteristics of certain songs that resonate strongly within specific contexts.

## 4. Discussion

The use of music as a tool for managing attention is a frequent topic of discussion within r/ADHD, highlighting the perceived link between auditory stimulation and cognitive function in this population. This aligns with research exploring the potential of music to modulate attention, particularly within the framework of selective attention. Selective attention theory, which posits that individuals actively filter sensory input to prioritize relevant information, provides a useful lens for understanding how music might influence attentional processes. For individuals with ADHD, who often experience deficits in attentional control and executive functions [58], music may offer a means of enhancing focus by creating a structured auditory environment that filters distractions. Recent research has investigated the effects of background music on cognitive performance in ADHD, with some studies suggesting potential benefits for tasks requiring sustained attention and working memory [12]. While the precise mechanisms by which music influences attention in ADHD remain under investigation, the active selection and use of music to manage focus reflects a conscious attempt to regulate attentional resources.

The phenomenon of “stuck songs” (involuntary musical imagery or earworms) resonates with concepts of cognitive rigidity and perseverative cognition often observed in ADHD. These intrusive musical loops can be interpreted as a manifestation of attentional mechanisms becoming “locked” onto specific auditory stimuli, reflecting broader challenges in cognitive flexibility [59]. These attentional fixations can be considered a form of involuntary attention, where cognitive resources are captured by the salient stimulus despite attempts to disengage. From the perspective of emotion arousal theory, as explored by [60], and within the broader context of constructed emotion [61], these repetitive loops can serve as a form of emotional self-regulation. The predictable structure of a stuck song may provide a sense of control and reduce uncertainty during periods of heightened emotional arousal, offering a calming effect. Conversely, the stimulating nature of the music might increase engagement during low-arousal states. This dynamic interplay between music, arousal, and emotional experience is central to contemporary understandings of emotion. Studies on music and emotional regulation have highlighted the use of music for managing mood and coping with stress [62]. While direct research on earworms and ADHD remains limited, these recent findings offer valuable insights into the cognitive and emotional mechanisms that may underlie this phenomenon in individuals with ADHD.

The distinct characteristics of focus music and stuck songs offer crucial insights. focus music, often characterized by lower loudness and higher instrumentalness, appears designed for deliberate attentional modulation. These features support sustained engagement and minimize distractions. While older research suggested benefits of instrumental music for focus [63], more recent studies emphasize the importance of individual preferences and task demands [64]. Additionally, research demonstrates that distracting auditory stimuli, including background speech and music, can negatively impact cognitive performance, especially on tasks requiring focused attention [65].

In contrast, stuck songs, typically high in energy and loudness with repetitive patterns [37], seem to “hijack” attention, aligning with research on perseverative cognition [66]. Recent research on involuntary musical imagery has thoroughly explored the phenomenology, dynamics, individual differences, and musical features of earworms [67], suggesting that various cognitive and musical factors contribute to their “stickiness.” The repetitive nature and high arousal of these songs may contribute to their “stickiness” by engaging automatic attentional processes.

The strategic use of focus music can be further understood through the lens of mood regulation and mood congruence theory. While a strict interpretation of mood congruence suggests that individuals seek out stimuli that match their current emotional state, the selection of focus music often represents a more nuanced approach. Individuals experiencing attentional difficulties or frustration may intentionally select calming, instrumental pieces to induce a more positive or neutral mood, thereby creating a mood-incongruent effect aimed at enhancing cognitive performance. This active manipulation of mood through music suggests a conscious effort to optimize internal states for focused work, highlighting the proactive role individuals with ADHD may take in managing their attentional resources. This active selection process contrasts with passive mood congruence, where one might be more drawn to music that reflects their current state, whether positive or negative.

The analysis of lyrical sentiment and popularity within the three categories (focus music, stuck songs, and general purpose) reveals distinct patterns that shed light on the cognitive and emotional processes associated with music engagement. focus music tended to gravitate towards positive or neutral lyrical sentiment, suggesting that individuals seeking to enhance concentration often prefer music that does not evoke strong or distracting emotional responses. This aligns with selective attention theory as emotionally neutral lyrics minimize competition for cognitive resources, allowing for greater focus on the primary task. This preference also resonates with the strategic use of mood regulation, as discussed earlier. Individuals may actively select music with positive or neutral lyrics to create an internal state conducive to concentration, potentially counteracting negative emotions associated with attentional difficulties. This active selection process also aligns with mood congruence theory, not in the sense of seeking music that reflects their current negative mood, but rather in strategically selecting music to induce a desired positive or neutral state. The “general purpose” category, encompassing a broader range of musical discussions, displayed a wider distribution of lyrical sentiment and popularity, reflecting the diverse ways in which music is engaged with beyond the specific contexts of focus and involuntary musical imagery. Notably, stuck songs exhibited a tendency towards positive sentiment and high popularity. This suggests that songs that become “earworms” are often not only widely recognized but also carry positive or neutral emotional connotations. The high prevalence of positive sentiment in stuck songs can be interpreted through the lens of emotion arousal theory [60]. Positive emotions, often associated with moderate levels of arousal, may enhance attention and memory encoding, making these songs more likely to become “stuck” in memory. Furthermore, the popularity of these songs suggests a social component, where shared cultural exposure and positive social associations may contribute to their increased salience and memorability. The combination of positive sentiment, moderate arousal, and high popularity likely creates a potent combination that increases the likelihood of a song becoming an involuntary musical image.

The methodology used in this study, while comprehensive, has several limitations. First, the reliance on the pushshift archive for reddit data introduces potential biases, as deleted or removed posts and comments may have been disproportionately excluded. The preprocessing steps, including the removal of posts with fewer than five comments, may have inadvertently excluded low-engagement discussions. In the music analysis, the reliance on Spotify and Genius APIs meant that only tracks available in these databases were analyzed, leading to potential exclusions of niche or lesser-known music.

### 4.1 Implications and future research

The findings of this study underscore the intricate interplay between music, cognition, and emotion in individuals with ADHD, presenting several compelling implications. The preference for focus music, characterized by higher instrumentalness and positive valence, aligns with several theories. These frameworks suggest that music serves as a scaffolding tool, helping individuals manage attention and regulate emotional states. For individuals with ADHD, this strategic use of music may act as a compensatory mechanism for executive function deficits, including challenges in sustaining attention and managing impulsivity. Extending this analysis, neurocognitive studies should focus on how ADHD is associated with unique patterns of auditory processing, which may amplify music’s effectiveness in modulating neural activity tied to focus and emotional regulation. This could refine our understanding of how music interacts with ADHD-specific neural pathways.

These findings have significant interdisciplinary implications as well, particularly for music therapy, education, and technological interventions. Music therapists could draw on insights about stuck songs and focus music to design personalized interventions that cater to ADHD-specific needs. Similarly, educators might integrate music strategically into learning environments to support students with ADHD, using curated playlists to enhance concentration or reduce anxiety. Furthermore, technology developers could harness these insights to create adaptive music applications that dynamically adjust to an individual’s cognitive and emotional states, offering real-time support for focus and mood regulation. An interdisciplinary approach integrating neuroscience, psychology, and musicology will be essential in developing personalized music interventions that support cognitive and emotional adaptation.

Future research must pursue longitudinal studies tracking the evolution of musical interventions for ADHD, examining how musical preferences impact cognitive management strategies over time. Methodological innovations are crucial, requiring more sophisticated data collection techniques that capture the musical experiences of individuals with ADHD. The community-driven insight done in the present study complements clinical perspectives, offering valuable qualitative data that reflects real-world practices. However, cultural factors could influence music preferences, suggesting a need for cross-cultural studies to explore how ADHD-related music behaviors vary globally.

## 5. Conclusion

The dramatic rise in ADHD diagnoses and treatment has created an urgency to examine innovative, non-traditional approaches to symptom management. This study highlights the potential of music, particularly with specific audio features and lyrical sentiments, as a tool to improve focus, regulate emotions, and support ADHD symptom management in real-world contexts.

Reddit’s anonymous, open-forum nature enables individuals with ADHD to share candid narratives about their music experiences. This work utilized the integration of advanced tools like Gemini 1.5 Pro and LLAMA 3.1 to enhance the analysis by enabling precise categorization and extraction of music-related data from over 9215 tracks. Additionally, audio, and lyrical analyses deepened the understanding of musical preferences and emotional impacts on r/ADHD community.

Categorization of posts from r/ADHD into focus music, stuck songs, and general purpose types revealed distinct audio feature profiles. focus music was characterized by higher valence (positive emotional tone) and instrumentalness, suggesting a preference for uplifting and instrumental tracks that aid concentration. In contrast, stuck songs exhibited higher energy, acousticness, and danceability, reflecting their tendency to be vibrant and engaging, often aligning with the phenomenon of “earworms.” Selective attention theory, which highlights how individuals filter sensory input to prioritize relevant stimuli, contextualized the role of focus music in reducing distractions. Emotion arousal theory provided a lens to understand stuck songs as tools for involuntary attention capture and emotional self-regulation.

Correlation analyses revealed notable differences in the relationships between audio features across categories. For example, energy and loudness exhibited a strong positive correlation across all categories, while focus music showed a unique moderate negative correlation between valence and instrumentalness. These variations highlight how music preferences in ADHD contexts are shaped by the functional roles of audio features. Sentiment and popularity analysis of song lyrics indicated that focus music leaned toward positive sentiment, while stuck songs clustered around highly popular tracks with emotionally positive content. The analysis of lyrical sentiment showed focus music avoids emotionally distracting content, enhancing cognitive engagement, while stuck songs leverage emotional positivity and high popularity. These findings suggest that music’s emotional and cognitive impact varies significantly across contexts, emphasizing its multifaceted role and potential for personalized ADHD management strategies. The implications from this work span multiple disciplines, offering practical applications in therapy, education, and technology.
